# Emotion, Aging, and Decision Making: A State of the Art Mini-Review

**DOI:** 10.20900/agmr20230003

**Published:** 2023-03-31

**Authors:** Joseph A. Mikels, David B. Taullahu

**Affiliations:** Department of Psychology, DePaul University, 2219 North Kenmore Avenue, Chicago, IL 60614, USA

**Keywords:** aging, emotion, cognition, decision making, intuition

## Abstract

Over the past few decades, interest has begun to surge in understanding the role of emotion in decision making, and more recently in studies across the adult life span. Relevant to age-related changes in decision making, theoretical perspectives in judgment and decision making draw critical distinctions between deliberative versus intuitive/affective processes, as well as integral versus incidental affect. Empirical findings demonstrate the central role of affect in various decision-related domains such as framing and risk taking. To situate this review within an adult life-span context, we focus on theoretical perspectives in adult development regarding emotion and motivation. As a result of age differences in deliberative and emotional processes, taking a life-span perspective is critical to advance a comprehensive and grounded understanding of the role of affect in decision making. Age-related shifts in information processing from negative toward positive material also have consequential implications. By taking a life-span perspective, not only will decision theorists and researchers benefit, but so too will practitioners who encounter individuals of various ages as they make consequential decisions.

At no time more striking than the present—as the world emerges from the pandemic of the past several years—we all face consequential decisions. These decisions encompass myriad life domains and involve risk and uncertainty. For instance, we all have faced the decision of whether or not to get the COVID-19 vaccination and possible booster shots. These decisions can have dramatic effects on all of our lives, but especially so for older adults [1–3]. Decades of theoretical and empirical work in judgment and decision making have revealed many intriguing insights. In more recent years, burgeoning theoretical and empirical interest has illuminated how affect impacts decision making [4,5]. Much of this work, however, has neglected consideration of how various processes may differ across the adult life span—though many of the concepts are linked to areas of age-related decline and/or growth. We will first provide a brief overview of the role of affect in the field of judgment and decision making, followed by a review of age-related changes in emotion and motivation relevant to decision making. Finally, we will consider how these age-related changes impact decision making across the adult life span – a now more prevalent state-of-the-art approach in theoretical and empirical investigations [6].

## THE ROLE OF AFFECT IN JUDGMENT AND DECISION MAKING

The behavioral and social sciences—including descriptive decision research and theory—have uncovered countless significant phenomena through the examination of decision processes among younger adults. Descriptive decision approaches characterize how people typically make decisions as opposed to how people should optimally make decisions. A descriptive approach opens an important vantage point to examine age differences in decision making, as opposed to age-invariant optimal decisions. Critically, descriptive findings from younger adults may not generalize to older adults [7]. Thus, it is critical to expand the scope of such work across the adult life span as changes in motivation, emotion, and cognition emerge as we age [6]. Historically, decision science has mostly focused on the deliberative aspects of decision making. However, there has been an affective revolution in the field with a groundswell of theoretical and empirical interest in understanding how emotions impact decision making [4]. Consideration of affect and decision making often draws from dual process models, which make the distinction between intuitive and deliberative processing [5,8–10]. Intuitive processes are experiential and generally quick, affective, and gist based. Deliberative processes, in comparison, are generally slow, analytic, and verbatim based. Although these distinctions have been incredibly useful, they have been criticized as somewhat oversimplified and too broad [11]. Thus, more integrative perspectives may be necessary.

As an example, a critical distinction useful in guiding investigations of affect and decision making involves considering when affect is integral versus incidental. Integral affect arises from the decisions, options, and/or outcomes that one currently faces, whereas incidental affect is unrelated to the choice at hand and can carryover from a previous situation [12,13]. This distinction describes how integral affect can be used as important information in guiding a decision [14,15]. Negative feelings about a risky gamble (i.e., integral affect) may wisely lead to a risk avoidant decision. However, positive feelings about a beautiful sunny day (i.e., incidental affect) may inadvertently lead to choosing a risky gamble, despite potential negative outcomes. In other words, the carryover of incidental affect can bias the decision at hand, resulting in a bias toward either increased or decreased risk perception and behavior. For example, different discrete emotions have been shown to influence risk estimates; people who are generally happy or angry make more optimistic judgments, whereas fearful people make more pessimistic judgments [12].

A prominent approach to considering the role of integral affect in decision making is the affect heuristic [16]. This theoretical framework explicates the beneficial role of integral affect in decision making, explaining how decision options are “tagged” with positive and negative affect [17–19]. Accordingly, the use the affective impressions in this “affect pool” can be simpler than deliberating over the decision attributes. The affect heuristic has been useful in explaining how easy or difficult it is to evaluate given attributes [20], how sensitive people are to framed proportion [21], how people make probability estimations [22], and how focusing on affective reactions in highly complex decisions can result in better decisions [23].

Integral affect has also been useful in considering the underlying mechanisms of prominent and robust biases in decision making, framing effects. Framing effects pertain to the observation that people make different choices depending on how probabilistically equivalent alternatives are described [24–26]. Framing effects can take various forms [27], such as message framing, which seeks to influence judgments and behavior and follows a pattern generally consistent with the affect heuristic. For example, when the risk of side effects for medications are described in a positive gain versus negative loss frame (e.g., 85% of people did not experience nausea, versus 15% of people experienced nausea, respectively), risk perception is lower, positive affect is higher, and there is an increased likelihood to take the medication [28,29]. In contrast, risky choice framing concerns the choices people make between certain and uncertain (risky) options. People often demonstrate risk aversion with gain frames (e.g., choosing to retain $40 of $100 over a gamble with a 40% chance of retaining the entire $100), but risk seeking with loss frames (e.g., choosing a gamble with a 60% chance of losing the entire $100 over a certain loss of $60 from $100). Importantly, evidence supports a prominent role for affect in risky choice framing—even with respect to brain activations [30]. Specifically, when individuals made decisions that were consistent with the framing effect (i.e., risk aversion in gain frames and risk seeking in loss frames), greater activation in an emotional processing region, the amygdala, was observed. In contrast, when people made choices that were inconsistent with the framing effect, greater activation in a brain region associated with controlled deliberative processing, the prefrontal cortex, was observed. In terms of integral affect, when young adults relied on emotion to make their decisions, they were more likely to choose the risky gamble option [31]. Moreover, the immediate integral feelings individuals had toward their choices—relative to how they anticipated they would feel after making a decision – explained risk taking [32].

Underscoring the utility of framing, such methods have been expanded through message framing in various health domains with the goal of impacting behavior change. With this approach too, better understanding how affect plays a role could inform how best to tailor such messages. Message framining has been theorized to differently impact different health domains [25]. Specifically, highlighting the health benefits of various preventative behaviors (e.g., exercise, nutrition) with gain frames should increase such behaviors. In contrast, emphasizing the consequences of not engaging in detection behaviors (e.g., screening for breast or skin cancer) through loss frames should increase those behaviors. As revealed by several meta-analyses [33–35], these message framing effects are only partially supported with inconsistent findings that gain frames are more influential than loss frames for various preventative health behaviors. This mixed support suggests that the causal pathways from framing to behavior are more complicated and not as direct as assumed, involving a number of mediators such as attitudes, message perceived effectiveness, and behavioral intentions [36,37]. Despite the importance of these more cognitive mediators, affective reactions are emerging as critical components as well with gain frames eliciting positive feelings and loss frames eliciting negative feelings [37–39]. Furthermore, higher perceived message effectiveness is correlated with higher positive feelings for gain frames [38], which in turn result in more positive attitudes and higher likelihood to accept the information [37]. Thus, integral affect across multiple domains plays a central role in decision making.

From a related theoretical perspective, positive and negative affect can play an important role in shaping intentions for behavior. The theory of planned behavior posits that behavior exists as a function of intentions and perceived behavioral control, or the belief in one’s ability to carry out the behavior [40]. According to this theory, intentions are comprised of three key determinants: subjective norms, perceived behavioral control, and attitudes toward the behavior. Specifically regarding affect, attitudes refer to favorable or unfavorable appraisals of a behavior. Strong feelings regarding a behavior, whether positive or negative, consistently and significantly predict intentions [40]. Thus, decisions based on behavioral intentions have clear affective underpinnings through attitudinal processes.

These multiple theoretical perspectives all specify the various ways affect is critically involved in judgments, intentions, and decision making. As a result of adult age differences in affective processes, theoretical views on the role of affect in decision making must take into consideration how older adults may make different decisions than younger adults. To provide the necessary foundation with which to consider age differences in decision making and the underlying affective influences, we first provide a brief overview of age differences in emotional experiences and processes.

## AGE DIFFERENCES IN AFFECTIVE EXPERIENCES AND PROCESSES

To grasp the full extent to which affect influences decision making for older adults, one must first consider the impact of aging on cognitive function. As adults enter their later years, deliberative processing abilities (e.g., attention, reasoning, memory, problem solving) typically begin to decline [41,42]. When older adults must solve interpersonal problems, however, they demonstrate greater flexibility than their younger counterparts—particularly when the problems are emotionally-charged [43]. Affect thus appears to play a role in some cognitive endeavors, which becomes a discerning factor given research that shows emotional functioning remains stable or is even enhanced with age.

Consistent findings over the past several decades indicate that emotional experience and well-being is at least as good, if not improved, with age. On average, older and younger adults report similar levels of emotional intensity and emotional expressive behavior [44]. Yet, across studies, older adults in comparison to their younger counterparts report not only lower negative affect, but also higher or at least sustained levels of positive affect [45–48]. Strikingly, even during the COVID-19 pandemic, older adults experienced less stress, less negative affect, and were more likely to use effective agentic coping strategies relative to younger adults [49]. Recent research has taken into account age differences in global life satisfaction, and still finds support for this experience of heightened positive affect in older adults [50]. In addition, older adults report greater emotion regulation compared to younger adults on self-report measures [51,52], and also demonstrate intact emotion regulation abilities in experimental settings [53].

Furthermore, complementing this research on aging and positive affect, older adults demonstrate a differential prioritization of positive over negative information relative to younger adults, the age-related positivity effect [54,55]. This effect describes an age-related trend in which a disproportionate inclination toward negative information in early life shifts across adulthood toward the positive. This phenomenon has been observed across numerous studies examining different processes from attention and memory to decision making [56], and a meta-analysis on the positivity effect that included 100 studies revealed it to be a robust and reliable phenomenon [57]. Analyses combining the results from all 100 studies revealed a significant pattern such that older relative to younger adults showed an information processing preference for positive over negative information. Notably, the positivity effect has also been demonstrated in response to emotionally ambiguous stimuli [32,58,59]. These findings suggest that emotional functioning remains strong in later life, and that positivity figures prominently in how older adults experience and view the world relative to younger adults.

In light of the divergence between maintained emotional processes in the face of declining deliberative processes as we age, researchers have investigated the idea that older adults’ maintained emotional functioning may compensate for the deliberative declines experienced in their advancing age. For example, evidence shows that older adults demonstrate superior long-term memory for emotional compared to non-emotional information [60–62]. Furthermore, even though aging is associated with declines in cognitive working memory, affective working memory remains intact even in old age [63]. Given that working memory is a central component of information processing [64], age differences in working memory have pertinent relevance on the decision-making process—particularly as decision complexity increases [65].

Multiple theoretical accounts address these changes in emotion across the life span. Socioemotional selectivity theory (SST) [55], a life-span theory of motivation, offers one prominent perspective. The theory posits that when future time horizons are vast, such as in youth, one’s focus is placed on gathering resources, knowledge, developing social networks, and so forth. When future time horizons narrow, as in later life, one’s focus is on the present and emotionally and socially fulfilling goals. An outcome of this theory is the understanding that in their pursuit of emotionally meaningful experiences, older adults may strive toward the augmentation of positive affect and the reduction of negative affect [44,54]. Alternative theorical perspectives offer other mechanisms to account for these effects [66]. For example, other candidate age-related mechanisms include the diminution of cognitive resources leading to greater positivity [67], the selective use of emotional strengths that support emotion regulation and positivity [68], the balance of age-related strengths with increased physiological vulnerabilities, which can lead to positivity when relying on intact strengths but negativity when vulnerabilities are tapped [69], age differences in appraisal processes that lead to evaluations consistent with goals of optimizing emotional experience [70], in addition to others.

As some of these theorical accounts consider motivation, it stands to reason that older adults may be more motivated by emotional components of decisions and more easily influenced by positive rather than negative emotions, partially due to age differences in future time perspective. A recent study by Strough et al. [71] found, for example, that when individuals focus on limited time rather than future opportunities, they tend to report higher preoccupation with negative events. However, older relative to younger adults were less preoccupied with those negative thoughts, despite facing greater perceptions of limited time. For aging populations in the modern era, making sound decisions is more pertinent than ever before. Thus, given the theoretical perspectives and empirical findings regarding emotion and decision making, as well as age differences in emotion, understanding the role of emotion for aging decision makers is essential.

## EMOTION AND DECISION MAKING IN LATER LIFE

Given these age differences in affective (and deliberative) processes, one can discern that older adults make decisions differently relative to younger adults. For example, when considering the role of integral affect, one study examined the use of deliberative versus affective strategies [72]. When decision strategies involved holding in mind and deliberating over details of the decisions, decision quality was improved for younger adults but impaired for older adults. Conversely, when decision strategies involved focusing on emotional reactions to decision options when making decisions, age differences disappeared; when employing affective strategies, the decision quality of older adults was equally as high compared to younger adults. From the broader dual-process perspective of deliberative versus intuitive processing, evidence from other studies indicates that intuitive processes do indeed benefit the decision making of older adults [73–75].

When considering the age-related positivity effect, older adults attend to and recall more positive versus negative decision information than younger adults [76,77]. Additionally, older adults have been shown to offer more positive versus negative attributes when evaluating choice options, resulting in increased choice satisfaction [78]. Furthermore, related work has shown that older relative to younger adults who focused more on positive rather than negative information when making a decision, reported higher positive mood and higher decision satisfaction levels afterwards [79]. Hence, for decisions that draw on integral affect, the age-related positivity effect appears to benefit decision satisfaction. However, by attending to and recalling more positive versus negative information, older adults might miss critical negative information and ultimately make non-optimal decisions. Notably, though, when the information being reviewed is for a consequential personally relevant decision, older adults focus on both the positive and negative information [79].

The age-related positivity effect is also evident in myriad instances of framing effects. For example, in the domain of risky choice framing, Mikels and Reed [80] found that whereas the decisions of both older and younger adults were equally biased by gain frames, the decisions of older adults were not biased by loss frames. With respect to health message framing, older adults better remember and assign higher informative value to positive gain versus negative loss framed health-related messages relative to younger adults [81]. Moreover, Notthoff and Carstensen [82] found that for older adults, gain-framed messages were more effective in increasing walking, whereas framing did not influence the walking of younger adults. Relatedly, older versus younger adults looked less at negative information about skin cancer, but in the end took more protective measures [83]. Though these findings are notable, what are the specific underlying mechanisms for these effects?

We propose that affective reactions play a particularly prominent role in age differences in framing. As described earlier, loss frames evoke negative feelings, whereas gain frames evoke positive feelings. Notably, though, the relationship between greater positivity for gain frames and greater perceived message effectiveness was stronger for older relative to younger adults [38]. Importantly, though, greater positive affect but not perceived effectiveness has been shown to influence the health decision behavior of older adults [84]. Collectively, these findings indicate that messages which evoke positive integral affect are effective for older adults in the health domain, and that the positivity effect may be beneficial to—or at least not impede—older adults’ health behaviors. Such findings regarding the influence of integral affect are consistent with the theoretical accounts, such as the theory of panned behavior.

Regarding incidental affect, though, some studies point toward harmful influences on the decision making of older adults. For example, von Helversen and Mata [85] found that older adults performed worse than younger adults when making sequential decisions. Interestingly, although deliberative processing ability was unrelated to performance, incidental positive affect was related to reduced performance. Specifically, increased positive affect appeared to result in searching through fewer options, and thus resulted in poorer performance. Similarly, in another study, incidental positive affect—but not declines in deliberative abilities—led older versus younger adults to make more non-optimal choices [86].

Given age-related changes in affective, motivational, and cognitive processes, research is starting to reveal age differences in risky decision making as well. First, though the literature is mixed, research generally indicates that risk taking propensity decreases relatively linearly from young adulthood through midlife into older age [87–93], with the results likely dependent on domain and methodology [6,94]. Additionally, positive affect appears to be particularly impactful in decision contexts for older relative to younger adults. For older adults, positive affect improves decision making via improved gain-loss learning [95]. Positive affect also plays a particular role in the risk taking of older adults, such that the positive affect that they anticipate they would feel from potential positive outcomes predicts risk taking [96]. In contrast, for younger adults, the negative affect that they anticipate they would feel from potential negative outcomes predicts their risk taking. Moreover, older adults are more willing to choose risky options after positive versus neutral and negative mood inductions than younger adults [97]. These findings suggest that affect potentially leads older adults toward greater risk taking. However, when choice options contain certain losses, negativity may come to play a larger role for older adults [98]. For example, relative to younger adults, older adults prefer certain gains over uncertain possible larger gains (risk avoidance) but prefer uncertain possible larger losses over certain smaller loss (loss avoidance) [99]. The particular salience of losses in later life may underlie a different motivational orientation for older relative to younger adults that focuses on avoiding losses as opposed to acquiring gains [100,101]. Thus, affect plays a central role in age differences in risk taking as well, but these effects require further consideration of gains versus losses.

## CONCLUSIONS

Affect undeniably plays a critical role in decision making, and that role clearly differs for older versus younger adults. Relying on intact affective processes instead of declining deliberative processes appears to have beneficial consequences for older adults. When older adults are making complex decisions, practitioners should allow older adults to consider their integral feelings in addition to carefully weighing all of details. However, in other instances, specifically when incidental affect may bias older adults, there may emerge problematic consequences. As such. practitioners should be careful that the decisions of older adults are not unduly influenced by unrelated feelings. These patterns indicate that researchers and practitioners need to strongly consider the influences of integral affective responses and incidental states on decision making across the adult life span—with an eye toward beneficial versus problematic outcomes.

In this review, we have underscored the distinction between integral and incidental affect intentionally. Taking the findings together, first, we propose that to compensate for their declining deliberative processes, older adults might benefit from using integral affect when making decisions under most circumstances. Second, we contend that incidental affect may be detrimental to the decision making of older adults. Future research focusing on such distinctions promises to be fruitful and could propel our understanding of when affect is beneficial versus harmful to decision making—especially in the later years.

In sum, though still somewhat scant, to thoroughly understand the role of affect in decision making, research examining age differences is critical, and such considerations have sizable societal implications as we all grow older. By integrating a life-span perspective with theoretical and empirical investigations into the role of emotion in decision making, the field will uncover a more balanced, comprehensive, and accurate knowledge base. Ultimately, and arguably most importantly, approaching affect and decision making from a life-span perspective holds promise to better help people of various ages and especially older adults make decisions that can enhance their lives and wellbeing.

## DATA AVAILABILITY

Data sharing not applicable to this article as no datasets were generated or analyzed during the current study.
